# Implant Placement Post Maxillary Sinus Lift Using Osseodensification Concept: A Case Report

**DOI:** 10.7759/cureus.21756

**Published:** 2022-01-31

**Authors:** Anilkumar Rodda, Rekha R Koduganti, Harish K Manne, Pranavi Gullapelli, Laxmi Jaahnavi Devarampati

**Affiliations:** 1 Department of Periodontics, Panineeya Institute of Dental Sciences & Research Centre, Hyderabad, IND; 2 Department of Prosthodontics, Panineeya Institute of Dental Sciences & Research Centre, Hyderabad, IND

**Keywords:** diagnostic rvg, implant placement, osseodensification burs, maxillary sinus lift, cbct

## Abstract

Pneumatisation of the maxillary sinus is a roadblock to the successful placement of endosseous implants in the posterior maxilla. It is mandatory that the sinus has to be elevated to facilitate implant placement, for which the operating clinician should be well versed with the anatomy of the sinus to avoid intraoperative mishaps. Many techniques of sinus augmentation have been tried and tested with successful outcomes. This article presents a report of a 60-year-old female who had root stumps in relation to upper left first maxillary molar. She wanted an implant to replace the root stumps, however, on cone-beam computed tomography (CBCT) examination the sinus was pneumatised and required augmentation. The root stumps were extracted and after a six-month waiting window, she was treated with an indirect sinus augmentation procedure using Densah™ burs (Jackson, MI: Versah, LLC) after which an implant (5 mm x 8 mm) was placed with good osseointegration at three-month follow-up. The Densah™ bur facilitated sinus lift procedure is a good option for pneumatised sinuses with inadequate residual bone height.

## Introduction

Partially, edentulous patients have a problem managing with a removable prosthesis as they may hinder comfort, aesthetics, and speech. Implants have replaced removable dentures due to higher success rates and performance [1]. However, after tooth loss in the maxillary posterior region, pneumatization of the sinus is a very common phenomenon. Surgical sinus floor augmentation can increase the ridge height by approximately 3-5 mm, facilitating simultaneous implant placement [2]. The conventional sinus lift techniques made use of standard drilling protocol with burs for preparing the site for implant placement. Osseodensification is a novel technique, wherein highspeed densifying burs are used in increasing sizes to preserve and compact the bone as the sinus floor is being elevated [3]. It is a recent innovation that has the added benefits of preserving bone during osteotomy preparation, increasing the primary stability of the implant, which in turn helps in better osseointegration and success of the implant [4]. This case describes a crestal sinus lift done using the osseodensification burs followed by implant placement.

## Case presentation

**Figure 1:**
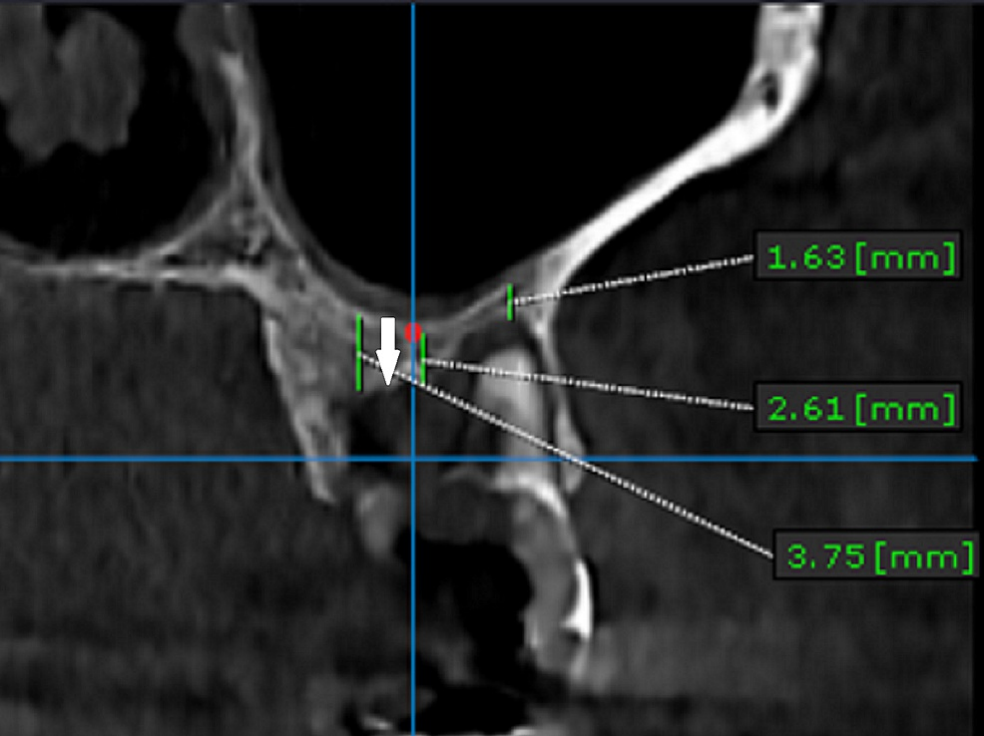
CBCT scan of the left upper first molar region taken preoperatively before the surgical procedure. The arrow denotes the distance from the sinus lining to the alveolar basal bone, and the distances are different at different points of the tooth involved. CBCT: cone-beam computed tomography

A 60-year-old healthy female visited the outpatient ward (OP) of a tertiary care referral hospital to have an implant placed in her left maxillary first molar region. A cone-beam computed tomography (CBCT) scan showed that there were root-stumps in that region requiring extraction; also, it was observed that the floor of the sinus was very close to the alveolar crest (Figure 1).

**Figure 2:**
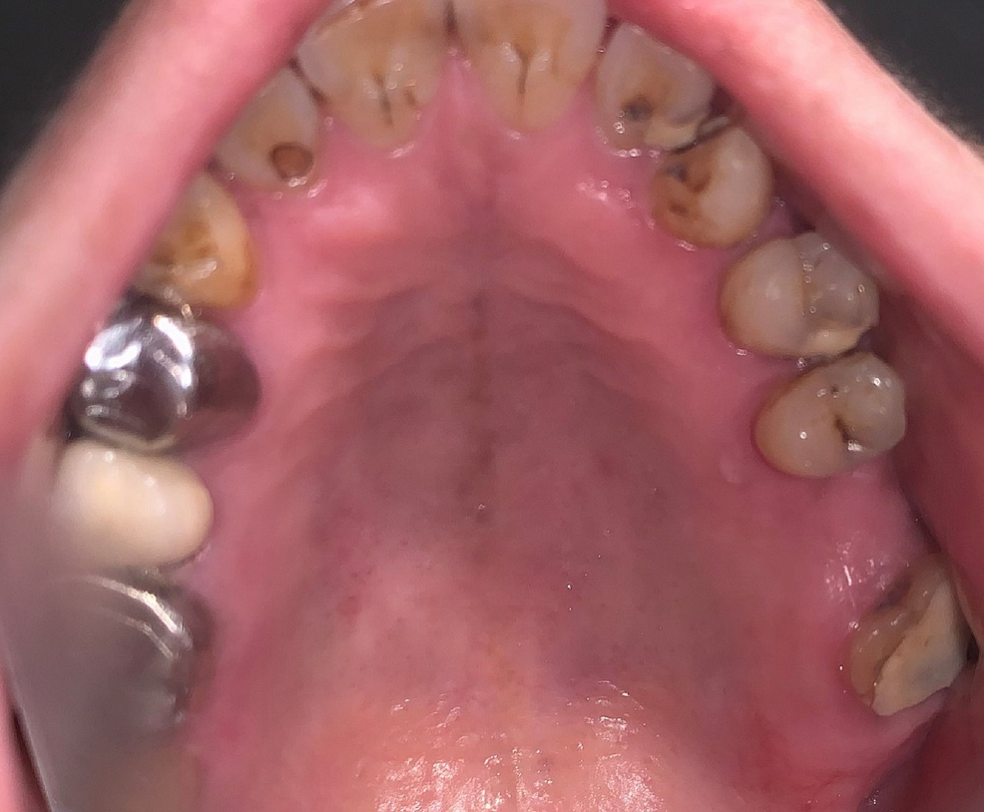
The clinical picture showing the edentulous site in relation to the left upper first molar, taken preoperatively

**Figure 3:**
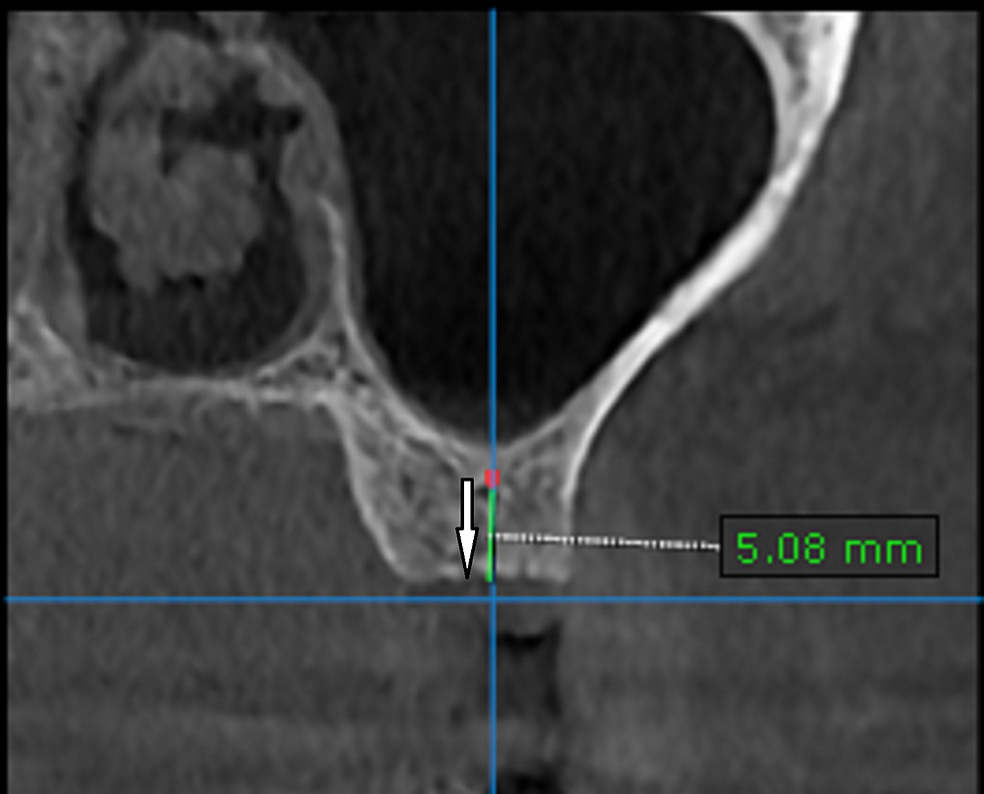
The CBCT scan of left upper first molar region taken three months after socket preservation with DFDBA. The arrow is depicting the increase in the bone volume after socket preservation. DFDBA: demineralized freeze-dried bone allograft

The root stumps were removed and the socket was preserved with demineralized freeze-dried bone allograft. Six months post-extraction, the patient visited the OP for implant placement (Figure 2). As the ridge height from the sinus floor to the alveolar crest was 5 mm, it was decided to go ahead with a crestal sinus lift to enable the placement of a 5 mm x 8 mm implant (Figure 3). Written informed consent was procured from the patient.

**Figure 4:**
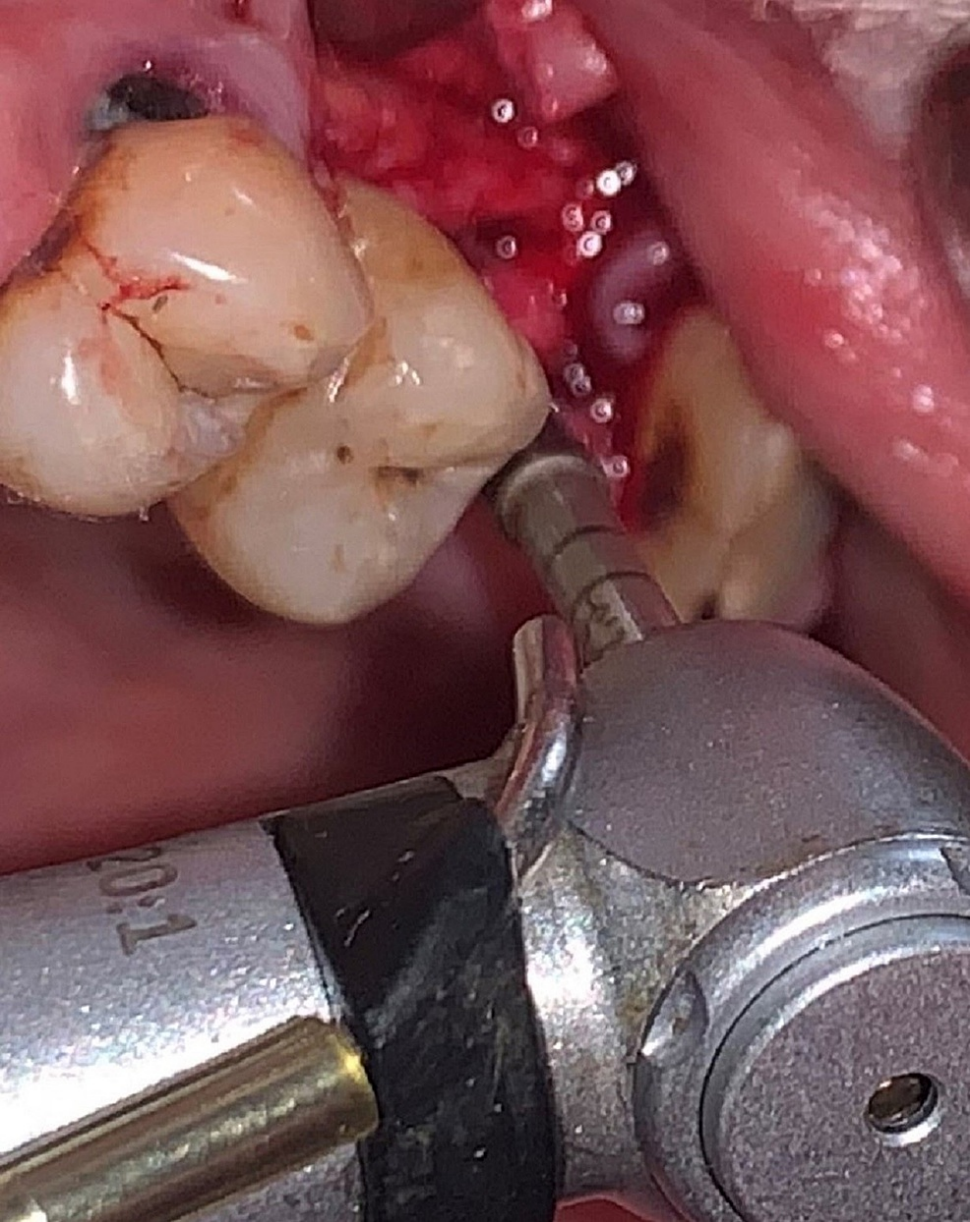
The intraoperative clinical picture showing the Densah bur being used for osteotomy preparation.

Then the flap was elevated using mid crestal and crevicular incisions. The osteotomy was started with the pilot drill in clockwise mode 1 mm short of the sinus floor. Then the osteotomy orientation in relation to the adjacent teeth was checked by inserting paralleling pin on radiovisiograph (RVG). Following this, a 2.5 mm Densah™ bur (Jackson, MI: Versah, LLC) at 800 rpm speed in an anticlockwise direction was inserted 1 mm short of the sinus floor (Figure 4). Sequential widening using 3 mm, 3.5 mm, and 4 mm was done, changing the drill motor to reverse-densifying mode with a gentle pumping motion following the manufacturer’s instructions. Additional vertical depth and membrane lift (in 1.0 mm increments) were achieved in the process. Intermittent pressure of one second off and on the bone, under copious saline irrigation was followed throughout the procedure. The final 4.5 mm drill was inserted only half the depth of the osteotomy to attain primary stability.

**Figure 5:**
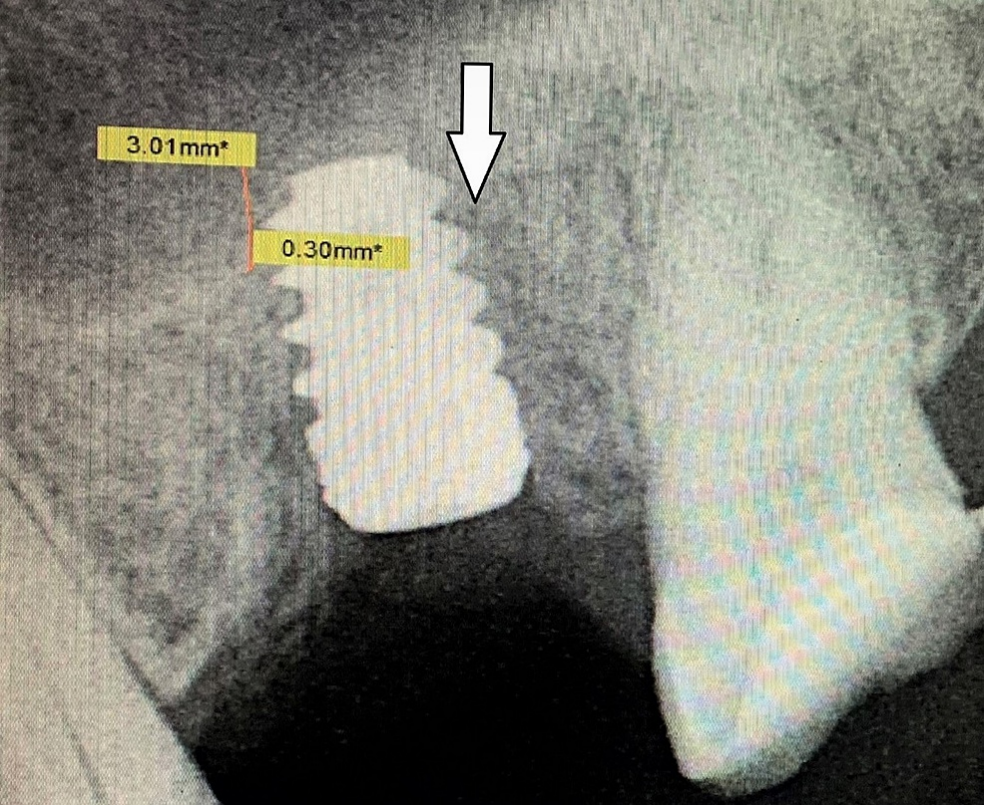
The image is showing RVG taken immediately after implant placement. The arrow is showing the amount of sinus lift (3.01 mm) achieved, by osseodensification burs, facilitating the placement of a 5 x 8 mm implant without traumatizing the sinus. RVG: radiovisiograph

An RVG taken immediately showed a lift of 3 mm thus facilitating placement of 5 mm × 8 mm implant (Figure 5). A cover screw was placed and the flap was sutured. Postoperative medication to help in healing included chlorhexidine mouthwash thrice a day, analgesics (aceclofenac 100 mg, paracetamol 325 mg), and serratiopeptidase (15 mg), and antibiotics (amoxicillin 500 mg) thrice daily for a week.

**Figure 6:**
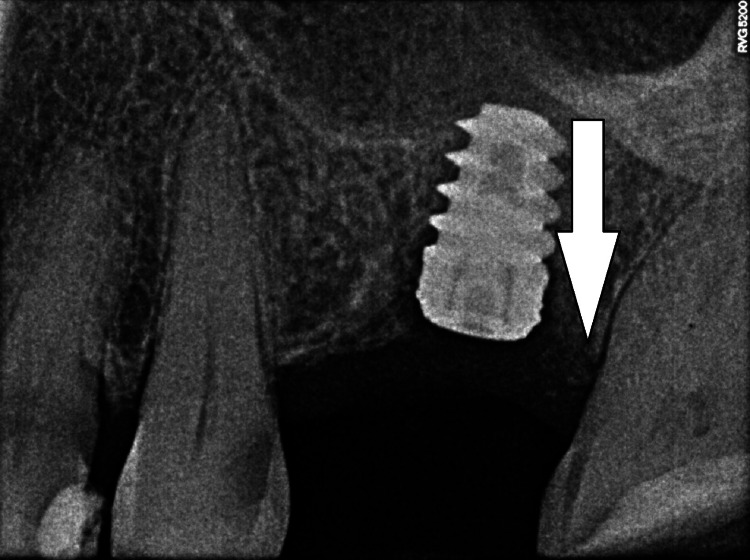
The image is showing RVG taken three months after implant placement. The arrow is depicting successful osseointegration of a 5 x 8 mm implant, three months postoperative. RVG: radiovisiograph

**Figure 7:**
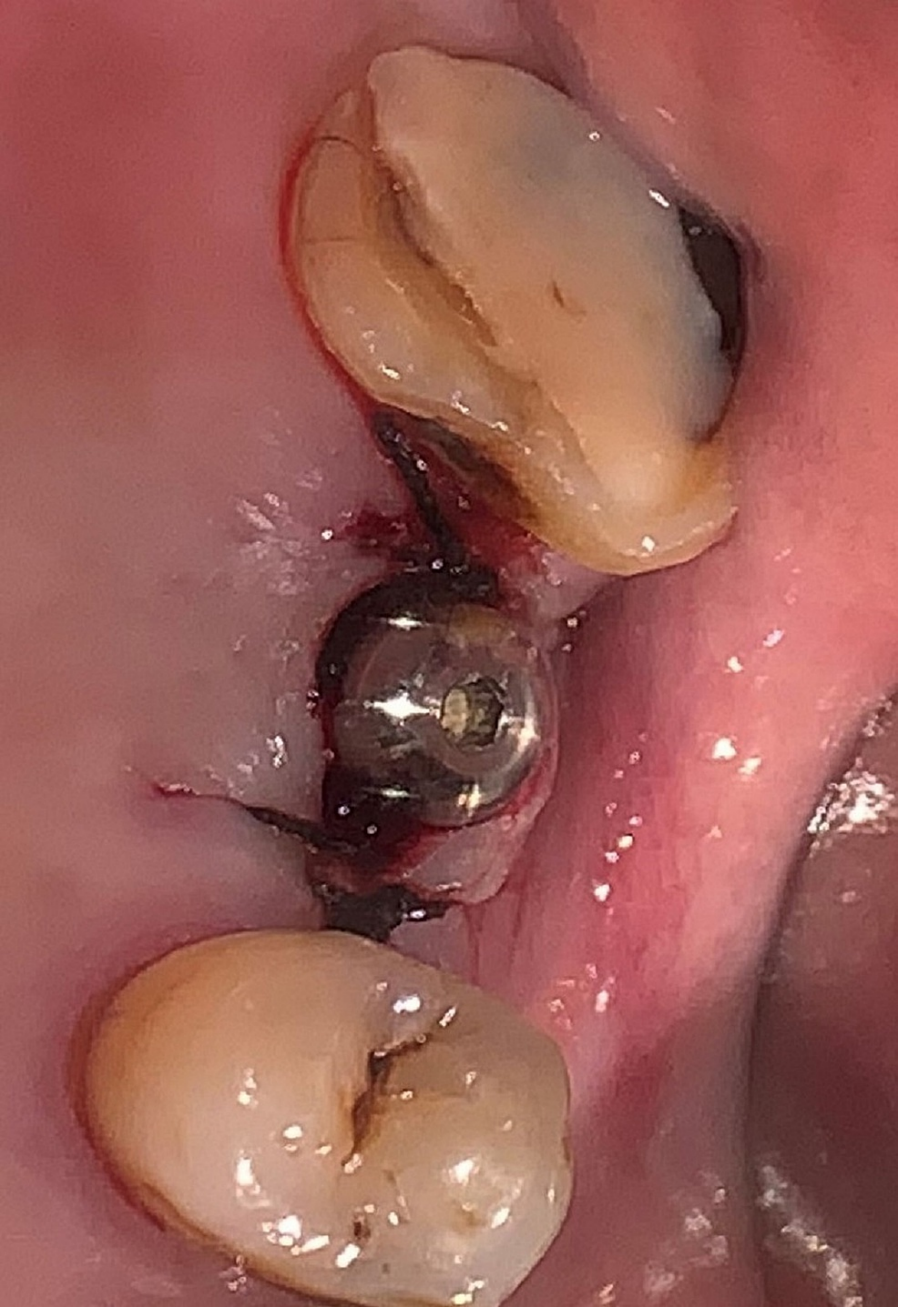
The clinical picture taken three months post-implant placement is showing the healing abutment tightened over the implant.

After three months, RVG showed good bone to implant contact (Figure 6). Hence the implant was exposed after flap reflection with a crestal incision. The cover screw was removed and healing abutment was tightened over the implant following which the flap was sutured back (Figure 7).

## Discussion

Boyne was the pioneer of the sinus augmentation procedure, following which, many indirect sinus augmentation protocols were proposed, including bone condensation by the use of osteotomes (summers-and its multiple variations), balloon lift, and hydraulic sinus condensing technique. But all these procedures carry a risk of paraesthesia, and Schneiderian membrane perforation, which cannot be ignored. Peri-implant bone microfractures were observed using Summer’s method which could hinder osseointegration [5]. It has been observed that parafunctional forces and smoking are risk factors for implant failure. The other causes are improper surgical technique, poor density of bone, and poorly controlled diabetes [6]. Trials are underway on the placement of shorter implants with surface modifications to alleviate the need for sinus floor augmentation in the posterior maxilla. A study was conducted on 14 patients who received Conelog® Screw-line implants (Basel, Switzerland: Camlog Biotechnologies GmbH) with a length of 7 mm. A total of 30 implants were placed and were assessed both clinically and radiologically, five years after loading of the implants. It was observed that there was 100% implant survival with an absence of peri-implant infections. The researchers thus concluded that short implants are a reliable option to replace missing teeth in the posterior maxilla avoiding sinus augmentation procedures [7]. Achieving proper bone to implant contact (BIC) is mandatory for implant stability and success. Prime factors for osseointegration include bone density, implant geometry, fastidious surgical planning, and proper patient selection. The conventional osteotomy preparation protocol revolves around removing the bone to suit the implant being placed, thus reducing the insertion torque leading to poor primary stability and potential lack of integration [3]. The novel osseodensification approach on the other hand uses burs with numerous grooves and increasing diameters in the anticlockwise direction, which favorably reduces the implant bed preparation, thus increasing the stability of the placed implant. Because of the counter-clockwise direction of these burs, it is hypothesized that there would be autogenous bone compaction at the apical end which facilitates gentle lifting of the sinus membrane. Using the osseodensification burs for a sinus lift is a minimally invasive procedure that negates the placement of graft material after sinus augmentation. It has been reported in a very recent systematic review that there was an increase in BIC and bone area fraction occupancy (BAF) [4]. Moreover, osseodensification technique has been demonstrated to increase the bone volume percentage around dental implants inserted in atrophic bone, with respect to the conventional implant drilling techniques, thus reducing the micromotion and enhancing implant stability [8].

## Conclusions

This case report describes the benefits of using osseodensification burs (Densah™) for sinus augmentation as well as implant site preparation. This protocol is best followed when the preferred sinus lift is ≤ 3 mm. Osseodensification is a very promising minimally invasive tool for crestal approach of sinus augmentation. However, longitudinal studies with larger samples are to be done to assert its role in sinus augmentation procedures.

## References

[1] Misch CE (2015). Rationale for dental implants. Dental Implant Prosthetics. Second Edition.

[2] Pjetursson BE, Lang NP (2014). Sinus floor elevation utilizing the transalveolar approach. Periodontol 2000.

[3] Huwais S, Meyer EG (2017). A novel osseous densification approach in implant osteotomy preparation to increase biomechanical primary stability, bone mineral density, and bone-to-implant contact. Int J Oral Maxillofac Implants.

[4] Padhye NM, Padhye AM, Bhatavadekar NB (2020). Osseodensification -- a systematic review and qualitative analysis of published literature. J Oral Biol Craniofac Res.

[5] Munjal S, Munjal S, Hazari P, Mahajan H, Munjal A, Mehta DS (2015). Evaluation of specifically designed implants placed in the low-density jaw bones: a clinico-radiographical study. Contemp Clin Dent.

[6] Munjal S, Munjal S (2019). Short (5.0 x 5.0 mm) dental implant placement: a case report with 3 year follow up. Mod App Dent Oral Health.

[7] Lorenz J, Blume M, Korzinskas T, Ghanaati S, Sader RA (2019). Short implants in the posterior maxilla to avoid sinus augmentation procedure: 5-year results from a retrospective cohort study. Int J Implant Dent.

[8] Trisi P, Berardini M, Falco A, Vulpiani MP (2016). New osseodensification implant site preparation method to increase bone density in low-density bone: in vivo evaluation in sheep. Implant Dent.

